# Revitalizing Astronomical Azimuth Determination: Integrating Modern Computing with Traditional Techniques

**DOI:** 10.3390/s25061871

**Published:** 2025-03-18

**Authors:** Luigi Barazzetti

**Affiliations:** Department of Architecture, Built Environment and Construction Engineering (ABC), Politecnico di Milano, Piazza Leonardo da Vinci 32, 20133 Milan, Italy; luigi.barazzetti@polimi.it

**Keywords:** automation, azimuth, observation planning, orientation, open-source, simulation, surveying

## Abstract

It is not widely recognized today that astronomical methods for azimuth determination continue to rank among the most accurate techniques for establishing orientation. However, their importance has diminished with the rise of GNSS techniques, which do not require clear skies and work in various weather conditions. Unlike GNSSs, astronomical methods rely on clear visibility and involve calculations based on spherical trigonometry. This work aims to simplify these calculations by providing open-source Python scripts that automate the process. These scripts also simulate the achievable precision, allowing users to plan optimal observations in advance. One of the aims of this work is to demonstrate that, in certain cases, the integration of ‘contemporary’ tools with techniques now regarded as ‘outdated’ can produce very accurate results. Beyond their purely historical significance, which has profoundly influenced modern surveying methods, the integration of astronomical measurements with modern computing codes allows surveyors to achieve azimuths with an accuracy of ±1–2 arcseconds.

## 1. Introduction

### 1.1. General Framework

Orientation to the north is widely recognized as a standard practice across numerous fields and applications due to its ability to establish a consistent and common reference direction. This alignment simplifies the interpretation of spatial data and facilitates communication among diverse users. Why specifically the north as a reference? Addressing this question is beyond the scope of this work, as it would require considering numerous historical aspects that extend beyond purely technical factors. Readers are referred to insightful books such as [1,2]. Instead, this work takes as its starting point the fact that the north has become an established reference, both in various technical disciplines and beyond, on an international level.

The concept of the north direction is fundamental in both historical and modern applications, serving as a key reference in navigation and cartography. This idea has been integral to the development of various disciplines throughout history and remains crucial in contemporary practices. For instance, in navigation, north orientation is essential for determining direction and plotting courses. Before the advent of the GNSS (global navigation satellite system) and other advanced navigational technologies, sailors primarily depended on celestial navigation to traverse the oceans [3,4,5]. Celestial navigation is deeply linked to astronomical measurements and spherical trigonometry, which have been fundamental tools for surveyors. Its enduring significance highlights the continuity of this concept across time.

North orientation is an essential element in modern disciplines ranging from geospatial analysis and urban planning to engineering and architectural projects, such as digital documentation. Orientation plays a critical role in archaeology, influencing the documentation, analysis, and interpretation of archaeological sites, artifacts, and landscapes. True north holds a fundamental role in archaeoastronomy [6]. Including a north arrow in plans is a common and standard practice. This simple yet essential symbol provides a consistent reference for interpreting the orientation of the depicted features. Nowadays, maps are almost universally oriented with north, which serves as a standardized framework for spatial analysis. When using modern GNSSs (global navigation satellite systems), north orientation provides a natural and universal basis for spatial reference, ensuring consistency in the collection, analysis, and presentation of geographic data. Astronomy, geospatial sciences, geography, geophysics, remote sensing, environmental sciences, etc.—the list of disciplines where this convention has become a standard practice is too extensive to cover exhaustively.

In surveying and mapping, north orientation is fundamentally related to the concept of azimuth, as both are integral to defining directions. North (particularly true north) serves as the fixed reference from which azimuths are calculated. Azimuth is defined as the angle measured clockwise from the meridian to the point of interest on the horizon. Different types of meridians are used to define azimuths, each suited for specific purposes. These include astronomic, geodetic, and grid meridians [7]. Converting between these azimuths requires understanding the specific relationships related to the Earth’s shape and map projections. Specifically, the Laplace equation enables the conversion of azimuths between geodetic and astronomical azimuths [8]. Meridian convergence, also known as grid convergence, allows for the conversion between geodetic coordinates and grid coordinates [9].

Historically, the concept of azimuth is closely tied to both astronomy and field astronomy in the context of surveying [10]. The practical connection between astronomy and surveying emerged from the fundamental need to establish positions and directions in the field. Surveyors working on the ground traditionally used astronomical observations to orient their measurements and create reliable reference points. Astronomical measurements have played a fundamental role in the development of modern surveying techniques, laying the foundation for contemporary surveying techniques and providing the methods and concepts that continue to guide measurement and mapping today. Before the advent of modern technology like GPS (Global Positioning System) or other GNSSs, surveyors relied heavily on star observations to determine positions and directions on Earth.

However, it is not widely known today that astronomical methods for azimuth determination remain some of the most accurate techniques available. At the same time, these methods directly provide true north, offering a unique advantage over other modern techniques. While satellite-based technologies have revolutionized surveying practices, the accuracy and reliability of astronomical observations continue to be unparalleled in certain contexts. Overall, this method is not only valuable for historical reasons but also holds practical significance in specific applications requiring high-precision surveying.

**Figure 1:**
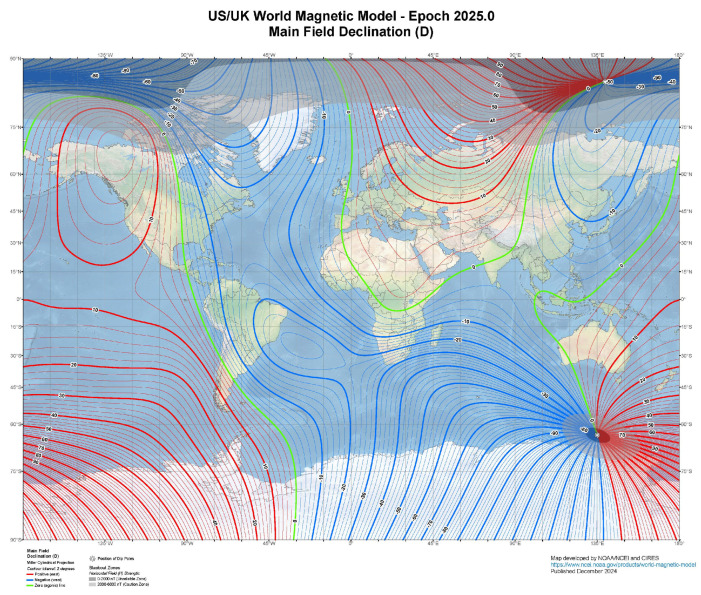
A compass can provide a general indication of the magnetic north direction, but when precise orientation to true north is needed, corrections for magnetic declination must be applied. In some regions, these corrections can reach values of 20° or more. The figure was derived from data available through the National Centers for Environmental Information (NCEI) World Magnetic Model, accessible at https://www.ncei.noaa.gov/products/world-magnetic-model, accessed on 5 March 2025.

Before moving on to the next section addressing the scope of this work, one final consideration deserves mention. In this manuscript, *magnetic azimuth* is not addressed, as the focus is on ‘true’ north orientation and azimuths measured relative to true north. Magnetic azimuths, which are based on magnetic north, can be measured using a compass, but they typically have lower precision (ranging from 1° to 5°) compared to other surveying methods. Since magnetic north differs from true north due to magnetic declination, a correction must be applied to obtain directional measurements relative to true north. The declination value varies by location and time, making it crucial to account for this difference when converting magnetic azimuth to true azimuth. The World Magnetic Model (WMM) provides a global representation of the Earth’s magnetic field and is used to calculate magnetic declination and other geomagnetic parameters worldwide [11]. The correction for magnetic declination can vary significantly depending on geographic location and time, ranging from nearly 0° to more than 20° (Figure 1). In areas near the magnetic poles, such as parts of Canada or Russia, declination can be very high, reaching 30° or more.

### 1.2. Scope of This Work

While astronomical measurements were once crucial in surveying for determining position and orientation, the development of faster and more automatic GNSS solutions [12] has largely replaced the need for these traditional methods in modern surveying practices.

GNSS measurements provide highly accurate position data and, particularly with advanced techniques such as differential GPS (DGPS) and real-time kinematic (RTK), can achieve positional accuracies down to a few centimeters or even a few millimeters [13]. This level of accuracy in positions generally surpasses what can be obtained through traditional astronomical methods, especially for routine surveying tasks.

Determining azimuths using GNSSs can also be highly accurate when using specialized equipment and differential acquisition. Different GNSS methods can yield azimuths with very high metric accuracy, depending on the baseline length [14] and techniques used. However, traditional astronomical methods for determining azimuths, which rely on observations of celestial bodies, can be even more accurate, often achieving accuracies of a few arcseconds or even less than 1 arcsecond. These methods, however, require clear skies, careful observation, and advanced calculations, which can lead to errors if not performed with meticulous attention to detail. The reader is referred to [15,16,17,18,19,20,21] for a review and discussion on various methods for determining astronomical azimuths and their diverse applications across different fields.

**Figure 2:**
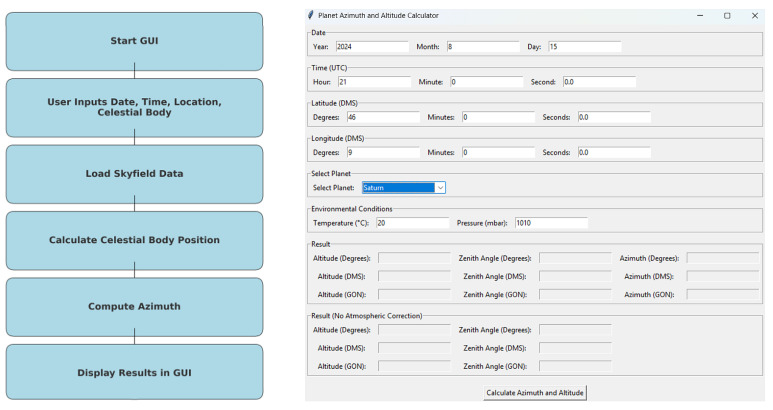
Simplified overall workflow and interface for planetary observation.

The main objective of this work is to simplify complex calculations by offering Python-implemented functions along with an intuitive graphical interface. This approach allows surveyors to make precise observations under favorable sky visibility conditions, achieving astronomical azimuths with accuracies within a few arcseconds, depending on the operator’s skill and the equipment used. The operator only needs a total station with a diagonal eyepiece, an application to obtain precise UTC time (which can be sourced from various mobile or desktop applications that use NTP servers or GPS time, or external radio-controlled clocks), and a stopwatch with a split function. Furthermore, Python’s open-source nature fosters collaboration and innovation, making advanced astronomical tools accessible to a wider audience and encouraging ongoing improvements. An image illustrating the overall workflow and interface for planet observation is presented in Figure 2. The script is a Tkinter-based GUI application designed to compute the azimuth and altitude of celestial objects. While azimuth is the primary focus, representing the horizontal angle between the observer’s meridian and the celestial body, the script also calculates altitude angles. These are crucial for astrogeodetic applications, including zenith angle determination. Furthermore, the program incorporates atmospheric corrections, adjusting altitude values based on temperature and pressure to enhance accuracy in real-world observations.

At the same time, the author would like to underline the historical importance of field astronomy for surveyors, an essential discipline that shaped the development of surveying techniques over time. Astronomical measurements involved the precise measurement of celestial bodies to determine coordinates and directions and establish reference points. This process was fundamental for experts, such as surveyors and cartographers, who relied on astronomical observations to map and chart vast territories. These methods laid the foundations for numerous disciplines, including physics, mathematics, statistics, calculus, astronomy, geography, engineering, navigation, and even environmental science, all of which have been critical to the advancement of science and technology.

The method described in this paper and facilitated by the Python scripts also holds practical significance for azimuth determination using basic surveying equipment. The work presented by [22] has already shown that highly accurate measurements can be achieved by observing Polaris, even with standard total stations (angle precision of 5–7 arcseconds).

**Figure 3:**
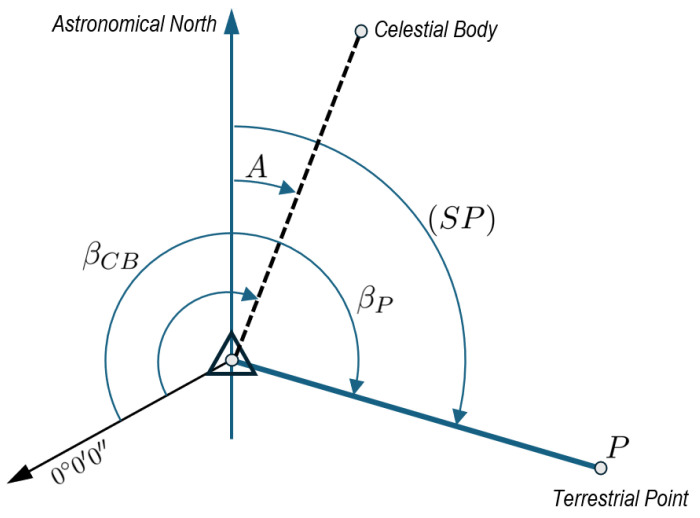
Scheme for calculating the astronomical azimuth. This basic framework can be easily adapted to other configurations using traditional reasoning about angles in classical surveying applications.

However, Polaris is only visible from the Northern Hemisphere. Near the equator, Polaris lies very close to the horizon, making accurate observation challenging. The implemented codes provide the azimuth of stars, four planets (Venus, Mars, Jupiter, Saturn), and the Moon. The user simply has to input the observation location (astronomical latitude Φ and longitude Λ) and UTC in the implemented graphic interface, obtaining the astronomical azimuth *A* of the selected body. Then, basic calculations using the horizontal circle readings $\beta_{P}$ taken with the total station with a (terrestrial) reference target *P* will provide the astronomical azimuth (SP) between the station point *S* and the target *P*. The user also needs the horizontal angle with the considered celestial body $\beta_{CB}$. Using the configuration shown in Figure 3, the astronomical azimuth can be calculated as $\left(SP\right)=A+\beta_{P}-\beta_{CB}$. This formula can be easily adapted to other configurations.

Stars are usually the primary choice for accurate measurements but, for the sake of completeness and considering the brightness of various celestial bodies (and the related visibility issues in specific areas, especially with significant light pollution), the code has been extended to include certain planets and the Moon. For example, Venus can reach a magnitude of −4.9, making it more luminous than any star. Jupiter, with a maximum magnitude of −2.9, also surpasses the brightness of most stars, including Sirius, which has a magnitude of −1.46. Mars can reach a magnitude of −2.9, comparable to that of Jupiter. Saturn, with a magnitude of around −0.5, is less bright but still exceeds the brightness of many stars. The Moon, however, is the most luminous, with a visual magnitude ranging from −12.7 to −13.5, significantly exceeding the brightness of any planet or star. The work of [23] demonstrated that the Moon can serve as a reliable alternative when stars are not visible.

The code is also suitable for planning observations, as it employs error propagation to estimate the uncertainty in the derived azimuth. This uncertainty arises from the input parameters’ uncertainties, specifically the hour angle $\sigma_{H}$ and latitude $\sigma_{\Phi }$. The uncertainty in declination $\sigma_{\delta }$ can be neglected, as the Skyfield library provides highly accurate celestial data, making the associated error negligible in our calculations. Indeed, the precision of the Hipparcos Catalogue is at the level of milliarcseconds, ensuring highly accurate positional data.

Accurate timing is essential for these measurements, as it significantly impacts the hour angle. The uncertainty in position is considered in this context because it is more practical and convenient to measure geodetic coordinates (ϕ,λ) using a GPS rather than taking complex astronomical measurements to determine astronomical coordinates (Φ,Λ). The difference between these two types of positions is the deflection of the vertical (ξ,η). If a geoid model is available, the correction can be applied as Φ=ϕ+ξ and Λ=λ+η/cosϕ. Different local geoid models are available on the International Service for the Geoid (ISG) webpage at https://www.isgeoid.polimi.it/Geoid/reg_mod.html, accessed on 5 March 2025. Global gravity field models can be found on the International Centre for Global Earth Models (ICGEM) website at https://icgem.gfz-potsdam.de/tom_longtime (accessed on 5 March 2025) which also offers a calculation service for computing the deflection of the vertical.

However, this process introduces uncertainties. The code allows for the inclusion of errors in $\sigma_{\Phi }$ and $\sigma_{\Lambda }$, representing the potential uncertainties in the astronomical coordinates derived from the geodetic measurements. The error in Λ directly affects the hour angle since the hour angle is determined by the difference between the observer’s local sidereal time and the right ascension of the celestial object. In this way, the user can simulate the error associated with the absence of a reliable correction for the deflection of the vertical, while also selecting optimal celestial bodies and the best times for measurement to minimize these errors.

A final consideration deserves to be mentioned before moving to the next sections. The Sun is not currently incorporated in this work, but the proposed code can be extended to include it for azimuth determination. The Sun offers the clear advantage of being visible during the day and has minimal parallax (approximately $9^{\prime \prime }$), which is manageable since the code already accounts for topocentric observations. However, using the Sun requires a solar filter for the total station, which typically costs around USD 200–300 (or more). Alternatively, a Roelofs prism would be an ideal solution for solar observations. As most users may not have this equipment, the current version of the Python code does not include solar observations, but this feature can be easily added in future updates.

## 2. Azimuth Calculation via Stellar Observation

### 2.1. Computation Strategy and Workflow

The implemented Python code, built using the Skyfield library https://rhodesmill.org/skyfield/ (accessed on 5 March 2025) calculates the azimuth and altitude of stars from Earth-based locations and times. It also incorporates environmental factors to provide a more reliable reading by correcting for atmospheric refraction [24]. To summarize the process, the program uses the observer’s location (latitude Φ and longitude Λ) and the current time to determine the star’s altitude and azimuth using spherical trigonometry. Azimuth measurements are generally less affected by atmospheric conditions compared to altitude. Skyfield provides an option to correct for atmospheric refraction. This correction applies only to altitude, not azimuth.

**Figure 4:**
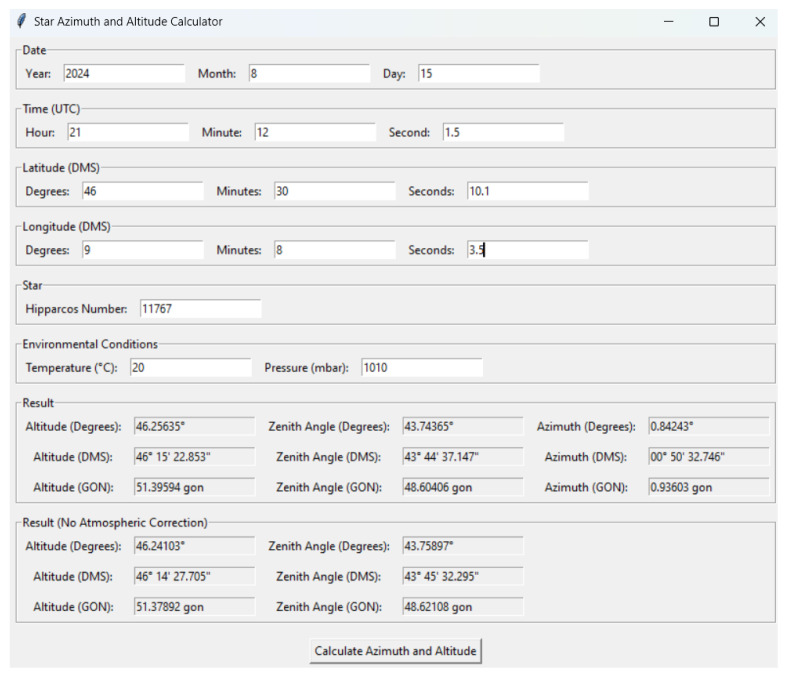
The interface in the case of stellar observations. The code also provides the star’s altitude but, in the context of this work, only the azimuth measurement is required.

The user inputs the date and time of observation, which are converted to UTC (Coordinated Universal Time) using Skyfield’s timescale system (Figure 4). Accurate timekeeping is essential for celestial calculations, as the position of stars changes over time due to the Earth’s rotation. The code accounts for the difference between UTC and UT1. The star is identified by its Hipparcos catalog number.

The process of determining a star’s azimuth and altitude from an observer’s position and time involves transforming the star’s equatorial coordinates into the observer’s local horizontal coordinate system. This transformation is necessary because, while celestial objects are typically described in the equatorial coordinate system, ground-based observations rely on the horizontal coordinate system (using azimuth and altitude).

In the equatorial system, a star’s position is given by the right ascension RA and declination δ. The first step is to calculate the star’s hour angle *H* to find the star’s azimuth and altitude at a given time. The hour angle represents how far the star has moved across the sky since it last crossed the observer’s local meridian. It is calculated by subtracting the star’s right ascension from the local sidereal time, which represents the right ascension currently on the observer’s meridian. The local sidereal time is computed based on the observer’s geographical longitude and the current UTC.

Once the hour angle is known, the code calculates the star’s altitude both with and without atmospheric correction. The code allows users to input environmental conditions and applies a model of atmospheric refraction to adjust the star’s apparent altitude.

While the calculated altitude is important, the primary focus of this work is the value of the azimuth, which can be estimated using the following relationship:

(1) $$A=\arctan\left(\frac{\sin(H)}{\cos(h)\cdot \sin(\Phi )-\tan(\delta )\cdot \cos(\Phi )}\right)$$

This equation accounts for the observer’s latitude Φ, the star’s declination δ, and the hour angle *H* to determine the star’s horizontal position. Once the altitude and azimuth are available, the program can convert these angles into different formats. For example, in addition to displaying the results in decimal degrees, the program converts them into degrees–minutes–seconds (DMS) format and gons.

### 2.2. Evaluating Azimuth Precision from Stellar Observations: Sources of Error and Implications

Error propagation is used to highlight how the observer’s latitude and the hour angle contribute to uncertainties in determining azimuth. Another Python code was implemented to understand the errors in azimuth determination introduced by latitude $\sigma_{A_{\Phi }}=\partial A/\partial \Phi \cdot \sigma_{\Phi }$ and hour angle $\sigma_{A_{H}}=\partial A/\partial H\cdot \sigma_{H}$ uncertainties and how these errors manifest in the calculated azimuth. While both latitude and hour angle affect the calculation of azimuth, the error propagation from each of these quantities is handled separately. The total azimuth error would be a combination of the errors due to latitude and hour angle. In addition, an error in the observer’s longitude $\sigma_{\Lambda }$ directly translates into an error in the hour angle, since the hour angle is defined by the observer’s local sidereal time, which depends on longitude. A small change in the observer’s longitude Λ is thus equivalent to a small change in the hour angle *H*, leading to a corresponding azimuth error.

The implemented function takes as input a Hipparcos catalog star ID and loads the star’s right ascension and declination. It also loads the ephemeris generated by NASA’s Jet Propulsion Laboratory (JPL), which provide the positions and velocities of Solar System bodies. The current implementation of the function calculates the errors based on the observer’s location and the position of a star at the current time. While this provides accurate results for the specific moment of the function’s execution, the function can be extended to account for specific times.

The latitude and hour angle errors are defined in arcseconds using the default values $\sigma_{\Phi }=20^{\prime \prime }$ and $\sigma_{H}=20^{\prime \prime }$. The error considered in the function is sufficiently large to account for a large deflection of gravity. If geodetic coordinates are measured using a GPS, a local geoid model can assist in determining the deflection of the vertical. This enables a more precise correction and a better estimate of astronomical coordinates, particularly in regions with significant topographic variations, where the deflection of gravity may introduce non-negligible discrepancies. The user can, of course, modify the code to input their own data, allowing them to adapt the numerical simulation for a more realistic simulation.

The function generates two separate plots. The first plot shows the azimuth error as a function of the hour angle due to latitude uncertainty. For each latitude, a line representing the error across the hour angle range is plotted. The second plot presents the azimuth error due to hour angle uncertainty, again plotting the error for each latitude across the range of hour angles. A range of hour angles (from −90° to +90°) is defined, corresponding to different positions of the star in the sky relative to the observer’s meridian. Additionally, four different latitudes (10°, 30°, 50°, and 70°) are considered to understand how the azimuth error varies with the observer’s location on Earth. The open-source nature of the code allows users to adapt the results to their specific needs, such as customizing outputs for a particular latitude.

**Figure 5:**
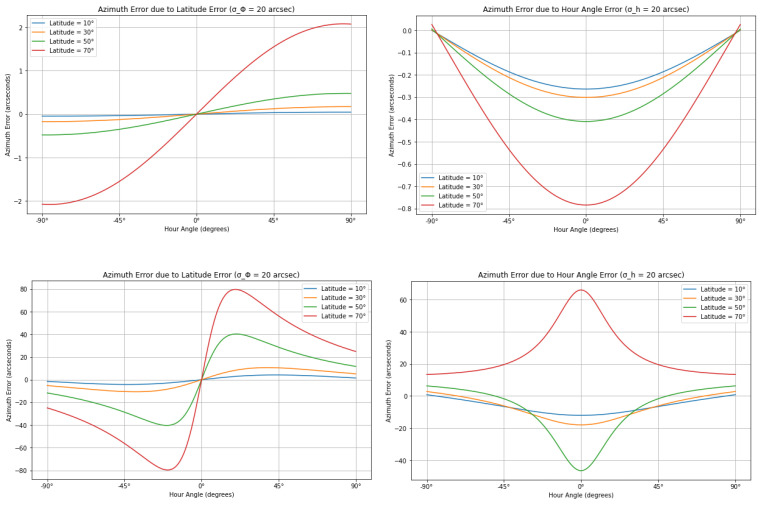
Comparison of Polaris (**top row**) and Dubhe (**bottom row**), assuming similar input errors.

A comparison between Polaris and Dubhe is shown in Figure 5. It can be an example of how the implemented code can assess precision for different stars. The azimuth errors are generally small for Polaris, which is near the celestial pole. The hour angle errors for Polaris show small variations, with the maximum error reaching less than 1 arcsecond. These errors are symmetric, with the largest deviations occurring near ±90° hour angles, and they remain relatively small across all latitudes. This behavior is expected given Polaris’s position near the North Pole, where its azimuth changes slowly over time. Latitude errors for Polaris result in slightly more significant deviations, though still modest compared to Dubhe. The maximum azimuth error due to latitude uncertainty reaches around 2 arcseconds. This error increases as the hour angle approaches 90° and is more pronounced at higher latitudes. However, despite these increases, Polaris’s overall azimuth error remains relatively minor. This also means that time measurement using Polaris does not require the precision of a high-accuracy chronometer (to tenths or even hundredths of a second). The total station’s internal clock, if properly synchronized to UTC time, can already provide an uncertainty on the order of 1 s. This level of precision is sufficient to automatically store angular and time observations in the instrument’s memory.

Dubhe, positioned much further from the celestial pole, exhibits significantly worse precision. The hour angle error for Dubhe is substantial, reaching up to 60 arcseconds near a 0° hour angle, especially at higher latitudes. Unlike Polaris, Dubhe’s azimuth is sensitive to changes in hour angle, particularly for observers at high latitudes. Similarly, the latitude errors for Dubhe are much more pronounced, with azimuth errors peaking at around $80^{\prime \prime }$.

The main difference between the two stars lies in the magnitude and behavior of their azimuth errors. Polaris, being near the celestial pole, exhibits much smaller azimuth errors overall, whether due to hour angle or latitude uncertainties. Its slow movement across the sky leads to a more stable azimuth. In contrast, Dubhe shows significantly larger azimuth errors, particularly when hour angle and latitude uncertainties are introduced. These errors are more pronounced at higher latitudes and near large hour angles. The greater sensitivity of Dubhe’s azimuth to hour angle and latitude reflects its position further from the pole, where its motion across the sky is faster and more susceptible to small observational inaccuracies.

**Figure 6:**
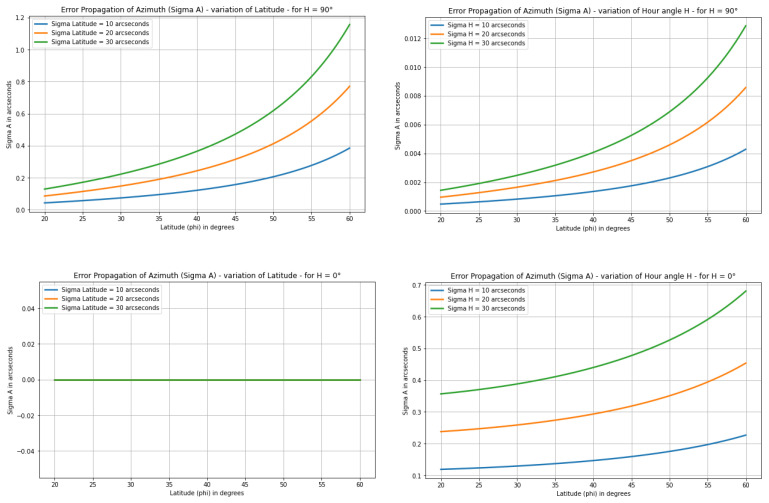
Precision for Polaris at various latitudes and different input error values.

The findings for Polaris align with the results presented in [22], which discusses the determination of astronomical azimuths using the hour angle methods. The error graphs for Polaris at different latitudes, derived from a modified version of the proposed code, closely resemble those introduced in this work (Figure 6). Minor discrepancies are likely due to variations in the declination values applied.

For observers in the southern hemisphere, Sigma Octantis serves as the reference star for azimuth determination due to its position near the south celestial pole. However, Sigma Octantis poses distinct challenges: it is much dimmer than Polaris, with a magnitude of 5.5, making it hard to observe with the naked eye or through standard instruments, particularly in suboptimal viewing conditions.

As mentioned in the introduction, the code can be used to test the precision of various stars and determine which are best suited for azimuth measurements anywhere on Earth. By evaluating the azimuth errors caused by uncertainties in hour angle and latitude, the code allows for the selection of stars that provide the most stable azimuth measurements based on the observer’s location. This capability is especially useful for surveyors seeking optimal reference stars.

## 3. Azimuth Determination Through Solar System Planets and Lunar Observations

This work also deals with the determination of astronomical azimuth using the Moon and the visible planets of the Solar System. The planets considered in this study are Venus, Mars, Jupiter, and Saturn, selected based on the capabilities of a total station telescope with approximately 30 × magnification. Unlike stars, planets and the Moon exhibit significant apparent motion relative to the background stars. This motion is primarily due to their proximity to Earth and their orbital paths around the Sun or Earth.

For azimuth determination with a total station, the great distance of stars renders the parallax effect practically negligible. In contrast, the parallax effect is more pronounced for planets, though still relatively small due to their considerable distance from Earth compared to the Moon. For example, the parallax angle for Mars is typically less than 70 arcseconds, while Jupiter, being farther away, has an even smaller parallax angle, usually under 10 arcseconds. These small parallax angles may introduce minor discrepancies in azimuth determination, but they can be corrected. The parallax effect is most significant when observing the Moon, given its proximity to Earth. At an average distance of about 384,400 km, the Moon’s apparent position can vary noticeably depending on the observer’s location, with its parallax reaching up to nearly 1 degree.

Incorporating planets and the Moon into azimuth determination, alongside stars, offers advantages and makes the process more exhaustive by expanding the range of usable celestial objects in the sky. While stars have traditionally been used due to their fixed positions in the celestial sphere, their visibility could be limited by environmental factors such as light pollution, weather conditions, or their position relative to the horizon. Planets and the Moon, however, could provide additional bright and accessible reference points, increasing the flexibility of azimuth measurements. The planets, especially Venus, Mars, Jupiter, and Saturn, are typically brighter than most stars and can be observed during dawn, dusk, or even twilight hours, extending the window of time for making observations. The Moon is frequently visible during the day and night. It remains visible even in heavily light-polluted areas due to its brightness.

Despite the dynamic movements of the planets and the Moon, their positions are well known and highly predictable due to the precise understanding of their orbits. This predictability allows them to serve as reliable reference points for azimuth determination, complementing the use of stars. Skyfield handles both the Moon and planets with a high degree of accuracy, applying the necessary corrections to ensure accurate azimuth determination. For the Moon, Skyfield calculates its position by accounting for its significant parallax effect due to its proximity to Earth. These corrections ensure that the Moon’s position remains accurate. For the planets, Skyfield factors in their orbital paths, light time delay (the time it takes light to travel from the planet to the observer), and the effects of Earth’s motion. This combination enables precise prediction of planetary positions. Additionally, Skyfield applies the required corrections for the planets, ensuring that their apparent positions are reliable for azimuth determination.

**Figure 7:**
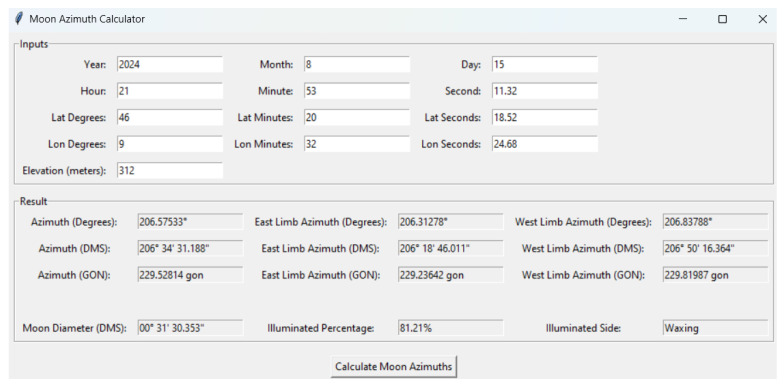
The interface in the case of lunar observations. The code calculates the diameter and provides the azimuth of the Moon’s east and west limbs, thereby enabling the observer to measure the visible limb.

The interface for planets closely resembles that used for stars. However, it includes an additional feature that allows the user to manually select from a predefined list of planets: Venus, Mars, Jupiter, and Saturn. In the case of the Moon, determining the azimuth for astronomical or surveying purposes can be challenging due to the difficulty of precisely measuring the Moon’s center. To overcome these challenges, the implemented code estimates the azimuth of both the east and west limbs of the Moon. This method uses the known diameter of the Moon and its predictable orbital motion, providing a more reliable means of calculating azimuth. The Moon’s angular diameter, which typically ranges between 29 and 34 arcminutes depending on its distance from Earth, is essential for determining the azimuth of both the east and west limbs. The code also calculates the phase angle of the Moon, which indicates whether the Moon is waxing or waning. Skyfield’s built-in functions also calculate the percentage of the Moon’s disk that is illuminated, providing additional information about the Moon’s current phase and brightness Figure 7.

For planets and stars, the code does not account for their angular diameters. Instead, it assumes that both planets and stars are observed from their central point, given their relatively small apparent sizes. Planets typically have angular diameters of just a few arcseconds. In this case, the user makes the observation when the vertical crosshair of the reticle is aligned with the center of the planet. Similarly, stars are treated as point sources due to their extremely small angular diameters, usually measured in milliarcseconds.

Different comparisons were carried out to validate the implemented code by using reference data produced by NASA’s JPL Horizons. The aim was to ensure that the code provides reliable numbers, maintaining accuracy and consistency in the results. As an illustrative example, a test was conducted for observations from a point located at latitude 46° and longitude 9°, recorded at 21:00:00 UTC for different years between 2004 and 2025, with a one-year step between observations. JPL Horizons is a system developed by NASA’s Jet Propulsion Laboratory (JPL). It provides precise data for various bodies within the Solar System. JPL Horizons integrates a variety of physical models, such as gravitational interactions, planetary perturbations, and relativistic corrections. These features make it one of the most accurate and reliable software systems available for generating reference observations. More information can be found on the official website: JPL Horizons (https://ssd.jpl.nasa.gov/horizons/, accessed 5 March 2025).

**Figure 8:**
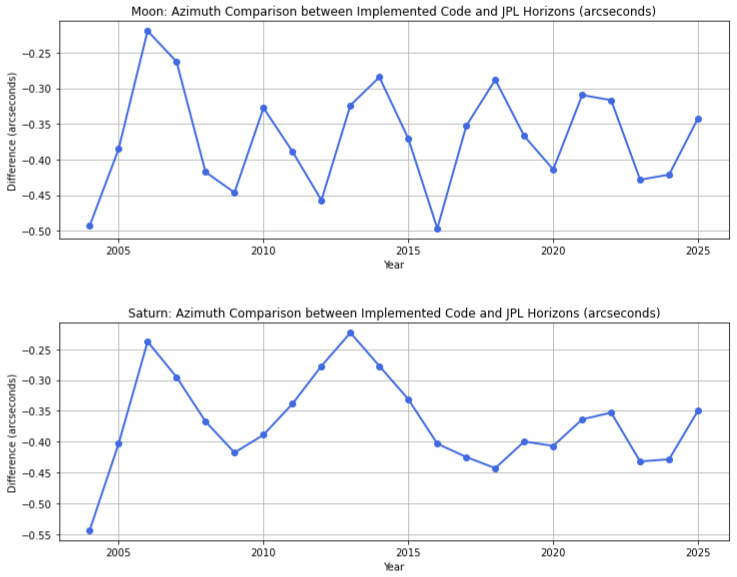
Comparison of azimuth values calculated using the proposed functions and JPL Horizons for the Moon and Saturn.

Plots for the Moon and Saturn are shown in Figure 8. The plots illustrate the difference between the azimuth values calculated using the proposed code and those from JPL Horizons. The small differences (less than 0.5 arcseconds) confirm the accuracy achieved by the implemented solution. These differences are within acceptable limits for the proposed azimuth calculations.

**Figure 9:**
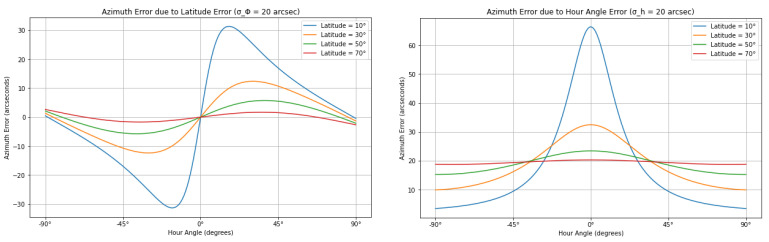
Simulation of observation precision using the Moon based on the specific time and location described in the manuscript.

As discussed in the previous section regarding stellar observations, the code can also be utilized to plot the precision achievable with observations of the Moon and planets. In these cases, the timing of the observation becomes more critical due to the rapid apparent motion of these celestial bodies. The Moon, in particular, exhibits significant changes in position across the celestial sphere within short time intervals, necessitating precise timing to ensure reliable estimates of achievable precision. While planetary observations also benefit from exact timing, they typically allow for a larger margin of error compared to lunar observations. The graph in Figure 9 illustrates the achievable precision using the Moon, considering the default values in the code, $\sigma_{\phi }=\sigma_{H}=20^{\prime \prime }$. The curves were generated for the date and time of 9 August 2024 at 19:30 UTC. In this scenario, the error is larger at lower latitudes, and the effect of the hour angle is more significant. Nevertheless, the curves demonstrate that the Moon can be considered a reliable target, especially if the input errors in positions and hour angle are minimized through precise modeling using a geoid model and accurate time measurement.

## 4. Field Work Measurements: A Real-World Example

The implemented codes were tested in various on-site campaigns. A representative result of the experiment is presented here to demonstrate the achievable accuracy. The experiment took place in Sondrio, Italy. The instrument used was a Leica TS30, with an angular precision of $0.5^{\prime \prime }$. The objective of the experiment was to measure the astronomical azimuth between a station point *S* and a reference terrestrial target *P* by observing multiple celestial objects, with measurements repeated over several days.

The positions of points *S* and *P* were also determined using static GPS techniques, allowing for the calculation of the geodetic azimuth, which was then converted into an astronomical azimuth using the Laplace equation. The creation of a highly accurate azimuth reference measurement via differential GPS required a long baseline. The points used are located in the city of Sondrio, where a permanent GPS station was also available and used as a fixed point for differential measurements. The distance between the selected points and the permanent station is less than 1 km. The static sessions enabled the calculation of the geodetic coordinates (ϕ,λ,h) for *S* and *P*, yielding a geodetic azimuth $\alpha =334^{\circ }59^{\prime }13.1^{\prime \prime }$ and a distance D=983.861 m, calculated using Vincenty’s formulas [25]. The corresponding astronomical azimuth was then derived using the Laplace equation:

(2) $$A_{G}=\alpha +\left(\eta \tan\phi +\left(\xi \sin\alpha -\eta \cos\alpha \right)\cot\zeta \right)=334^{\circ }59^{\prime }12.5^{\prime \prime }\;$$

The first term represents the azimuthal component of the DoV. The second term can be interpreted as an “error” in setting up the total station with the true plumb line rather than the ellipsoidal normal. When the deflection of the vertical becomes significant, this term grows stronger, especially with increasing values of cotζ. Consequently, this direction-dependent term is generally small, on the order of a few tenths of an arcsecond in flat areas, but it can reach some arcseconds in mountainous regions. The values of the deflection of the vertical were calculated using the Italian geoid model *ITALGEO05* [26,27] (values were computed on the geoid surface), resulting in $\xi =-14.8^{\prime \prime }$ and $\eta =-2.4^{\prime \prime }$.

An estimate of the expected accuracy for this reference angle can be made by assuming that the GPS coordinates were measured with a precision better than ±0.01 m, which corresponds to an angular error of $\left(0.01/D\right)\cdot 180/\pi \cdot 3600\approx 2^{\prime \prime }$, reinforcing the necessity of a long baseline.

The astronomical coordinates used in the code were calculated based on a static GPS solution, which was corrected for the deflection of gravity. Time was measured using a mobile application capable of receiving GPS time, along with a chronometer featuring a split function. The synchronization error was determined through multiple measurements and averaging the resulting error. A personal equation of 0.3 s was applied to the calculated UTC to correct the systematic observational error caused by individual variations in perception, reaction time, and manual operation during measurements [28].

Three measurement campaigns were carried out on different days, taking only a few measurements for each celestial body (between 6 and 12). The results are shown in Table 1, and can be directly compared looking at the computed values of the astronomical azimuth. As can be seen, values with Polaris in different days are very similar, with a difference of about $0.5^{\prime \prime }$. The reader may wonder why Polaris was not used during the second measuring campaign. The bad weather conditions, with a cloudy sky, made it impossible to see the North Star.

Polaris would, of course, always be the first choice under favorable weather conditions. However, this demonstrates that the method can also be applied using other stars. To provide a general idea of the relative ease of measuring Polaris compared to other celestial bodies, it was estimated that, during the first campaign on August 9, the azimuth of Polaris changed by approximately 0.2 arcseconds/s. In contrast, Dubhe moves at about 4.5 arcseconds/s, the Moon at about 11.1 arcseconds/s, and Altair at 16.4 arcseconds/s. Measuring these other bodies with precision clearly requires significantly more practice.

The results obtained with other stars are also quite good, with discrepancies of about 5–6″. These findings confirm the suitability of using other stars; however, users in the Northern Hemisphere with latitudes between 20 and 60 degrees should always prioritize Polaris as the first choice. The largest discrepancy was found with the Moon, showing a difference of approximately 30″, which is significantly worse than the results achieved with stars. This issue is mainly due to the difficulty in measuring the leading edge of the Moon, as opposed to the center of a star. This error could likely be reduced with further work on this celestial body, particularly in improving the precision of collimation.

**Figure 10:**
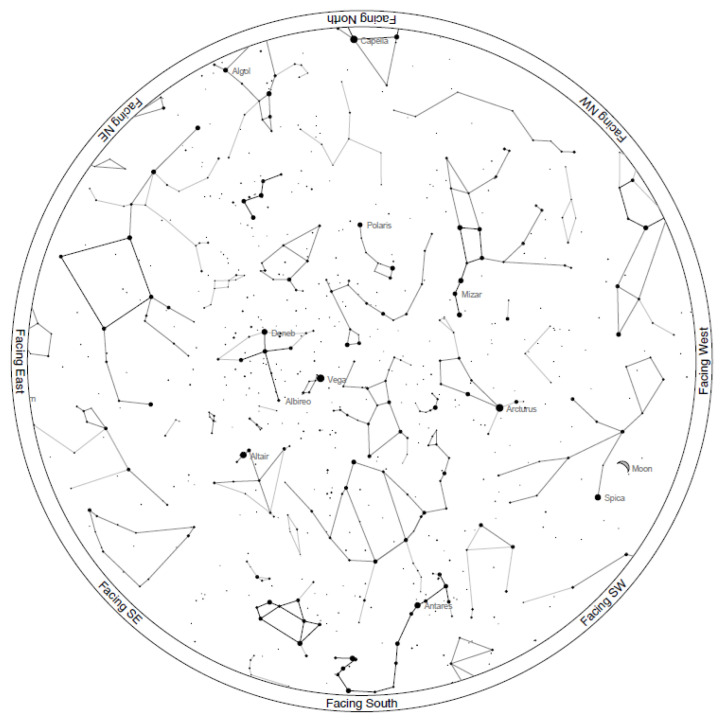
Sky chart generated using the free web service https://skyandtelescope.org showing the sky configuration during the first data acquisition in Sondrio on 9 August 2024, at 19:30 UTC.

A user without prior experience in astronomy might face challenges in identifying various stars or planets at night. A basic understanding of constellations is therefore essential. However, numerous mobile applications are available that allow users to point at different sections of the sky and instantly identify celestial bodies. The author believes that these applications make it possible for anyone to recognize stars and planets without prior experience. Additionally, observations can be planned in advance using free and open-source planetarium software, enabling the creation of sky charts tailored to a specific location and time. These charts can be printed for convenience, making data acquisition straightforward and efficient. An example of a sky chart generated using the free web service https://skyandtelescope.org (accessed on 5 March 2025). is shown in Figure 10. This chart corresponds to the sky configuration during the first data acquisition in Sondrio on 9 August 2024.

The astronomical azimuth derived from celestial observations is very similar to the value measured from the static GNSS survey corrected using the Laplace equation. It is important to note that the astronomical results were obtained with several short measurement campaigns of just a few minutes, simply by setting up the total station at the designated point and manually synchronizing the chronometer with a free application. In contrast, the GNSS required a longer acquisition time in static mode (about an hour and a half), as well as the additional steps of reaching the target point and setting up the antenna. The long baseline (nearly 1 km) contributed to the high precision of the GNSS-derived azimuth. However, such a long baseline is uncommon in standard surveying applications, as the second point must also be measured with a total station, which can introduce intervisibility challenges in areas with obstructions. The astronomical method is likely not only accurate but also faster, especially considering that calculations can be simplified using the provided code. The main limitation, however, remains environmental conditions—celestial observations are impossible under cloudy skies.

## 5. Conclusions

This study presented a Python tool for determining astronomical azimuth using celestial observations, offering a high level of precision comparable to or better than modern GNSS methods. The Python code simplifies the complex calculations required for azimuth determination and integrates the observation of stars, planets, and the Moon, thus extending the range of observable celestial objects. In addition to performing accurate calculations, the provided code can simulate achievable precision, enabling users to plan observations anywhere on Earth by estimating the expected accuracy based on location, time, and environmental factors.

The experiments demonstrated that, under favorable conditions, astronomical azimuths with an accuracy of ±1–2 arcseconds can be obtained within a few minutes, which surpasses the accuracy typically achieved by standard GNSSs in practical settings. Field tests validated the precision of the implemented methods. Observations using Polaris yielded consistently accurate azimuths, with a maximum deviation of only 0.5 arcseconds between campaigns. Other stars such as Altair and Dubhe also provided reliable results, though with slightly larger discrepancies. The Moon, despite its larger size and parallax, was shown to be a viable target, with a maximum error of approximately 30 arcseconds, though this can likely be improved with further refinement in collimation techniques. The achieved results for Polaris are consistent with those presented in [22], confirming that highly accurate azimuth determinations can be obtained using basic surveying equipment. However, the proposed version extends beyond Polaris, incorporating calculations for other celestial bodies. This broader approach allows for greater flexibility in astrogeodetic applications, enabling azimuth determination based on a wider range of celestial references.

The comparison with GNSS methods revealed that while GNSSs offer a reliable solution, particularly for long baselines, the astronomical approach is faster and less reliant on specialized equipment or extended measurement sessions. The main limitation of astronomical methods remains environmental, as clear skies are essential for successful observations.

The concept of revisiting outdated methods with a more contemporary approach, supported by the implementation of tools that assist the user, enables achieving a high level of accuracy without requiring extensive knowledge of the principles of astronomy and spherical trigonometry that historically defined the field of surveying. Undoubtedly, an understanding of these methods remains valuable for the modern surveyor, beyond mere historical curiosity.

In conclusion, the astronomical method, when combined with the provided Python tool, offers a robust, fast, and precise alternative to GNSSs for azimuth determination. The code’s ability to simulate precision further enhances its utility by allowing users to plan optimal observation strategies worldwide. Future improvements may focus on refining the accuracy of lunar observations. Moreover, integrating solar observations could further extend the tool’s applicability for daytime surveying tasks.

## Figures and Tables

**Table 1:** The computed astronomical azimuth in different days with different celestial bodies. This value can be compared with the geodetic azimuth corrected using the Laplace equation: $334^{\circ }59^{\prime }12.5^{\prime \prime }$; the difference is reported in the last column.

	*9 August 2024*		
Celestial body	Number of observations	Computed azimuth	Difference
Polaris	16	$334^{\circ }59^{\prime }11.1^{\prime \prime }$	$-1.4^{\prime \prime }$
Altair	8	$334^{\circ }59^{\prime }6.8^{\prime \prime }$	$-5.7^{\prime \prime }$
Moon	12	$334^{\circ }58^{\prime }44.1^{\prime }$	$-28.4^{\prime \prime }$
Dubhe	8	$334^{\circ }59^{\prime }15.4^{\prime \prime }$	$+2.9^{\prime \prime }$
	*29 August 2024*		
Celestial body	Number of observations	Computed azimuth	
Arcturus	8	$334^{\circ }59^{\prime }5.2^{\prime \prime }$	$-7.3^{\prime \prime }$
Alkaid	6	$334^{\circ }59^{\prime }5.9^{\prime }$	$-6.6^{\prime \prime }$
Cebalrai	8	$334^{\circ }59^{\prime }16.9^{\prime \prime }$	$+4.4^{\prime \prime }$
	*30 August 2024*		
Celestial body	Number of observations	Computed azimuth	
Altair	8	$334^{\circ }59^{\prime }10.7^{\prime \prime }$	$-1.8^{\prime \prime }$
Polaris	8	$334^{\circ }59^{\prime }11.7^{\prime \prime }$	$-0.8^{\prime \prime }$

## Data Availability

The data presented in this study are available on request from the corresponding author.
